# Roles of Autophagy in Elimination of Intracellular Bacterial Pathogens

**DOI:** 10.3389/fimmu.2013.00097

**Published:** 2013-05-06

**Authors:** Eun-Kyeong Jo, Jae-Min Yuk, Dong-Min Shin, Chihiro Sasakawa

**Affiliations:** ^1^Department of Microbiology, College of Medicine, Chungnam National UniversityDaejeon, South Korea; ^2^Infection Signaling Network Research Center, School of Medicine, Chungnam National UniversityDaejeon, South Korea; ^3^Department of Microbiology and Immunology, Institute of Medical Science, University of TokyoMinato-Ku, Tokyo, Japan; ^4^Department of Infectious Disease Control, International Research Center for Infectious Disease, Institute of Medical Science, University of TokyoMinato-Ku, Tokyo, Japan

**Keywords:** autophagic receptors, autophagy, innate immunity, *Listeria*, *Mycobacteria*, *Salmonella*, *Shigella*, xenophagy

## Abstract

As a fundamental intracellular catabolic process, autophagy is important and required for the elimination of protein aggregates and damaged cytosolic organelles during a variety of stress conditions. Autophagy is now being recognized as an essential component of innate immunity; i.e., the recognition, selective targeting, and elimination of microbes. Because of its crucial roles in the innate immune system, therapeutic targeting of bacteria by means of autophagy activation may prove a useful strategy to combat intracellular infections. However, important questions remain, including which molecules are critical in bacterial targeting by autophagy, and which mechanisms are involved in autophagic clearance of intracellular microbes. In this review, we discuss the roles of antibacterial autophagy in intracellular bacterial infections (*Mycobacteria*, *Salmonella*, *Shigella*, *Listeria*, and *Legionella*) and present recent evidence in support of molecular mechanisms driving autophagy to target bacteria and eliminate invading pathogens.

## Introduction

**Figure 1:**
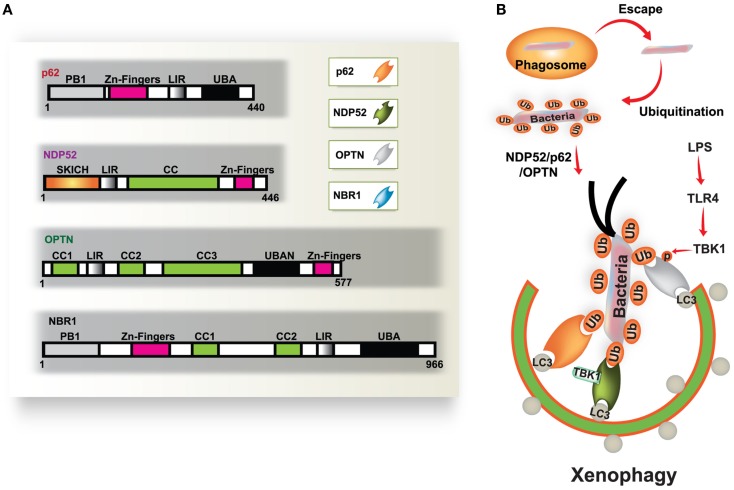
**Diverse cargo receptors are involved in the activation of selective autophagy**. **(A)** Schematic model of the autophagic cargo receptors p62, NDP52, optineurin (OPTN), and NBR1. These receptors interact with both ubiquitin on substrates and LC3 on the phagophore, which results in the activation of autophagy. **(B)** Xenophagy is induced by ubiquitinated substrates derived from various bacteria. Many intracellular bacteria, such as *Salmonella*, are sequestered and replicate within *Salmonella*-containing vacuoles (SCVs), but some bacteria that escape from SCVs are recognized and ubiquitinated for recruitment to p62, NDP52, and OPTN, which results in their transportation to the phagophore. NDP52 interacts with LC3C through its CLIR domain, inducing antibacterial autophagy. TBK-1 activated by TLR4 induces phosphorylation of OPTN at Ser177, which leads to enhanced binding affinity for LC3. Moreover, TBK-1 is involved in the activation of NDP52-mediated autophagy (right). CC, coiled coil domain; PB1, Phox and Bem1p domain; UBD, ubiquitin binding domain.

Autophagy is a fundamental protein degradation pathway essential for cellular homeostasis in response to various environmental and cellular stresses. The autophagy pathway is clearly involved in multiple aspects of innate and adaptive immunity (reviewed by Deretic and Levine, 2009; Virgin and Levine, 2009; Levine et al., 2011). During infection, a specific role for autophagy has been shown in the capture and degradation of intracellular bacteria and viruses, known as “xenophagy” (Levine, 2005; Deretic, 2011). In recent years, evidence of the specific roles of autophagy in selective targeting of bacteria through autophagic adaptors has accumulated. The main autophagic adaptors or receptors include; sequestosome 1 (SQSTM1/p62), nuclear dot protein 52 kDa (NDP52), optineurin (OPTN), and neighbor of BRCA1 gene 1 (NBR1) (Kirkin et al., 2009; Thurston et al., 2009; Mostowy et al., 2011; Wild et al., 2011; von Muhlinen et al., 2012; Korac et al., 2013) (Figure 1). These receptors function as cargo adaptors for the connection of substrates to the autophagy-related gene 8/microtubule-associated protein 1 light chain 3 (ATG8/LC3) family of proteins (Shaid et al., 2013).

Antibacterial autophagy plays an important role in controlling bacterial replication and promoting innate immunity in host cells. Increasing evidence has revealed that intracellular bacteria in vacuoles can be targeted by autophagy activation for lysosomal fusion and degradation (Levine, 2005; Deretic, 2011). Additionally, access to the cytosol for intracellular bacteria, caused by damage to the vacuoles, enables autophagy targeting of bacteria for eventual delivery to lysosomes (Ogawa et al., 2009; Collins and Brown, 2010; Fujita and Yoshimori, 2011). Several intracellular bacteria, including *Salmonella*, *Listeria*, *Legionella*, and *Mycobacteria*, can translocate their virulent components into the host cell cytoplasm. Moreover, these intracellular bacteria often induce the formation of ubiquitinated protein aggregates, which are recognized by cargo adaptors, and are ultimately destroyed by autophagy (Ogawa et al., 2009; Collins and Brown, 2010; Fujita and Yoshimori, 2011). More recent work has revealed the structural characteristics of the conserved interactions between cargo adaptors and the ATG8/LC3 family of proteins (Shaid et al., 2013). However, LC3 is not always necessary for recruitment of the autophagic membrane structure, and mechanisms for LC3-independent targeting remain to be explored (Noda et al., 2012).

In this review, we summarize recent data describing how autophagy and cargo receptors target important human pathogens. We focus on *Mycobacteria*, *Salmonella*, *Shigella*, *Listeria*, and *Legionella*, and the autophagy-mediated elimination of these intracellular bacteria.

## Antibacterial Autophagy in Mycobacterial Infection

**Figure 2:**
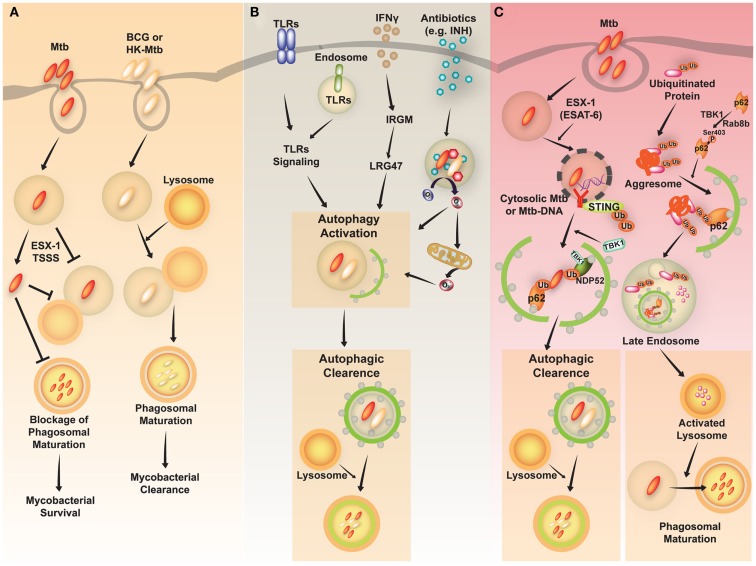
**A schematic diagram of *Mycobacterium tuberculosis* (Mtb) infection and clearance via the autophagic pathway**. **(A)** Mtb, but not *M. bovis* BCG, has diverse strategies for evading host immune system. Phagosomes containing Mtb do not fuse with lysosomes and mature into the phagolysosomes by preventing phagolysosome biogenesis. The restricted fusion of phagosomes with lysosome is attributable to limited entrance of lysosomal hydrolases to Mtb, preventing acidification of phagosomes. Mtb, but not *M. bovis* BCG or heat-killed (HK)-*Mycobacteria*, can evade to the cytosol depending on the ESX-1 Type VII secretion system. **(B)** Diverse stimuli including toll-like receptors (TLRs), interferon (IFN)-γ, and antimycobacterial antibiotics induce activation of the autophagic pathway to eliminate Mtb. The activation of endosomal and plasma membrane TLRs is linked to the induction of xenophagy of phagocytosed Mtb. IFN-γ induces autophagy through a downstream effector, Irgm1, in human macrophages, which then results in autophagic clearance of Mtb. Antimycobacterial antibiotics activate autophagy, which depends on cellular and mitochondrial reactive oxygen species. **(C)** The activation of autophagy plays a critical role in the clearance of intracellular *Mycobacteria* through diverse signaling pathways. *First*, ubiquitinated proteins are internalized and delivered via vesicles to the late endosome. Cargo receptors, such as p62, recognize ubiquitinated proteins and bind to LC3, contributing to autophagy activation. The autophagic vacuoles which contain ubiquitinated proteins traffic to the late endosome. This process promotes activation of lysosomes and fusion of Mtb-containing phagosomes with the lysosomes. *Second*, cytosolic recognition of Mtb-DNA via the STING-dependent pathway promotes ubiquitination of Mtb, and delivery of bacteria to autophagosomes through the cargo receptors p62 and NDP52. Finally, various stimuli induce autophagic clearance.

*Mycobacterium tuberculosis* (Mtb) is a successful human pathogen that survives in a phagosomal environment in mononuclear phagocytes after invasion by means of inhalation (Huynh et al., 2011; Harriff et al., 2012). Phagosomal compartments containing Mtb are known to evade fusion with lysosomes, thus arresting phagosomal maturation during mycobacterial infection, while nutrient delivery continues, enabling survival and replication of the bacteria (Vergne et al., 2004; Philips, 2008). Numerous bacterial proteins and lipid effectors are known to be involved in delaying the fusion of Mtb phagosomes with lysosomes, and in cytokine-dependent changes in phagosomal protein composition (Philips, 2008; Steinhauser et al., 2013). Despite the ability of Mtb to interfere with phagosomal maturation, an accumulation of evidence [including immunogold electron microscopy (EM) data] which shows that Mtb, but not *Mycobacterium bovis* bacillus Calmette-Guérin (BCG) can accumulate in the cytosol (van der Wel et al., 2007). Cytosolic translocation of Mtb depends on the 6-kDa early secretory antigenic target of Mtb (ESAT-6) Secretion System (ESX)-1 type VII secretion system, encoded in the region of difference 1 (RD1) of the Mtb genome, which has not been found in BCG or in heat-killed *Mycobacteria* (van der Wel et al., 2007) (Figure 2A).

As virulent Mtb strains can resist and inhibit autophagosome formation and its fusion with lysosomes (Deretic et al., 2006; Vergne et al., 2006; Deretic, 2008), divergent exogenous stimuli have been proposed to induce antibacterial autophagy targeting Mtb to inhibit its intracellular replication through enhancement of Mtb phagosomal maturation (summarized in Figure 2B). Autophagy activation via nutrient starvation, interferon (IFN)-γ, Toll-like receptor (TLR) stimuli, or by vitamin D treatment, has promoted phagosomal acidification and inhibited the survival of intracellular Mtb (reviewed by Deretic et al., 2006; Basu et al., 2012). In IFN-γ-induced mycobacterial xenophagy, LRG-47 (Irgm1; LPS-stimulated RAW 264.7 macrophage protein 47), a downstream effector of IFN-γ, plays an essential role in induction of autophagy and generation of autolysosomal organelles to inhibit intracellular mycobacterial replication (Singh et al., 2006). A recent study showed that bactericidal antibiotics activated the antibacterial autophagy process and contributed to successful antimicrobial responses during treatment for Mtb infection (Kim et al., 2012). This strongly implies that autophagy activation can overcome the Mtb-induced phagosomal maturation blocking process, and that it enhances host defense against Mtb. Several important questions remain to be answered, such as how to destroy Mtb in lysosomal compartments. Previous findings indicate that induction of autophagy in Mtb-infected macrophages promotes the delivery of ubiquitin conjugates to the lysosome, showing that at least one mechanism involving the generation of ubiquitin-derived peptides can enhance the bactericidal capacity of the lysosomal fraction (Alonso et al., 2007; Purdy and Russell, 2007).

The mechanisms by which Mtb phagosomes recruit autophagic machinery are also not fully understood. Recent studies have revealed that extracellular Mtb-DNA released from Mtb can be recognized by the stimulator of IFN genes (STING)-dependent cytosolic pathway, marked with ubiquitin, and delivered to the autophagic machinery through the selective autophagic receptors p62 and NDP52 (Watson et al., 2012) (Figure 2C, left). Importantly, the Mtb ESX-1 secretion system is critical for cytosolic sensing of bacterial DNA, and activation of the ubiquitin-mediated selective autophagy pathway in natural Mtb infection (Watson et al., 2012). Moreover, cytosolic sensing of Mtb-DNA is mediated through the STING/TANK-binding kinase 1 (TBK-1)/IFN regulatory factor 3 (IRF3) axis, and results in IFN-β secretion. Note that IRF3^−/−^ mice are protected from long-term Mtb infection, indicating that cytosolic sensing of Mtb-DNA and type I IFN signaling may contribute to the pathogenesis of tuberculosis (Manzanillo et al., 2012). Moreover, another study hinted at novel roles for Rab8b, a member of the Rab family member of membrane trafficking regulators, and TBK-1, with regard to autophagic elimination of *Mycobacteria* in macrophages (Pilli et al., 2012) (Figure 2C, right). TBK-1 phosphorylates the autophagic receptor p62, thus playing an important role in linking the innate immune response to cargo recruitment into autophagosomes (Pilli et al., 2012).

Other recent studies have shown that virulent Mtb inhibits autophagosome maturation in dendritic cells, and that this is dependent on the ESX-1 system (Romagnoli et al., 2012). The recombinant BCG and Mtb H37Ra strains with genetic complementation, using either the ESX-1 region from Mtb (BCG::ESX-1) or the PhoP gene (Mtb H37Ra::PhoP), a regulator of ESAT-6 secretion, restored their inhibitory activities against autophagy (Romagnoli et al., 2012). Classic autophagy activation by rapamycin treatment led to an increased interleukin (IL)-12 production and T helper cell (Th)1-oriented response in dendritic cells infected with Mtb (Romagnoli et al., 2012). These data partly correlated with previous findings in which mammalian target of rapamycin (mTOR) signaling negatively regulated the synthesis of IL-12 and IL-23 in human monocyte-derived macrophages infected with Mtb (Yang et al., 2006). These conflicting results are most likely due to the use of different cell types from different species (e.g., mouse or human), and variations of Mtb strains (e.g., Erdman strain, BCG, or others). Therefore, we must understand how antibacterial autophagy is activated in different cells and through which mechanisms. This information will help to identify and develop new therapies against Mtb infection.

## Antibacterial Autophagy in *Salmonella* Infection

*Salmonella enterica* serovar *typhimurium* (*S. typhimurium*) is a facultative intracellular pathogen with a bimodal life style inside host cells. The pathogen usually resides in a membrane-bound, *Salmonella*-containing vacuole (SCV). In this compartment, *S. typhimurium* can replicate and deliver a variety of effectors through type III secretion systems (TTSSs), allowing bacteria to enter the cytosol. SCVs can also develop into long tubular structures, also known as spacious vacuole-associated tubules, sorting nexin 3 (SNX3) tubules, and *Salmonella*-induced filaments (SIFs) (Bakowski et al., 2008; Schroeder et al., 2011). Some bacteria within damaged SCVs escape into the cytosol and can be detected by the autophagy process, which depends on the *Salmonella* pathogenicity island 1 (SPI-1) TTSS (Birmingham et al., 2006).

**Figure 3:**
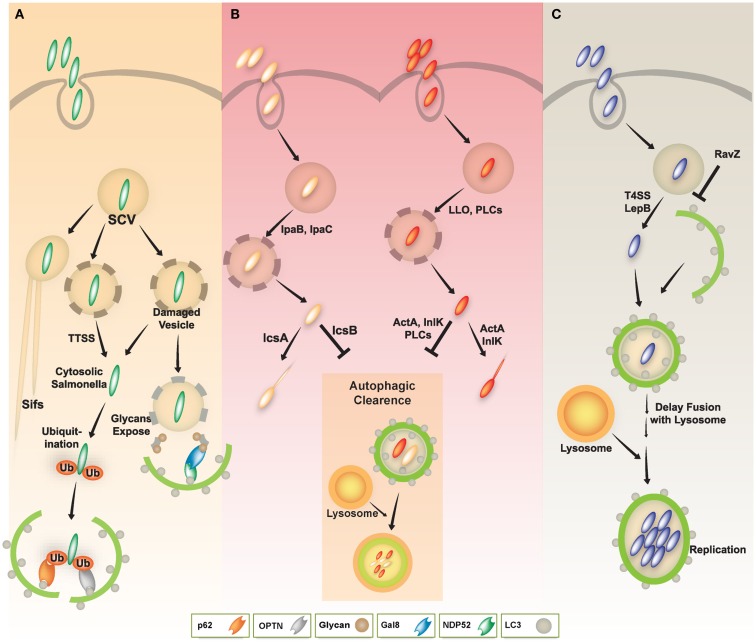
**A schematic diagram of diverse intracellular pathogen infections and clearance via autophagy pathway**. **(A)** In *Salmonella* infection, the majority of *S. typhimurium* resides in *Salmonella*-containing vacuoles (SCVs) and allow establishment of a niche permissive for growth, which then form *Salmonella*-induced filaments (SIFs). Some *Salmonella* enter the host cytosol via type III secretion system (TTSS). The cytosolic *Salmonella* via TTSS-dependent damage to the SCV was targeted by the autophagy system through ubiquitin-dependent or -independent pathways. The cytosolic *Salmonella* can become coated with ubiquitin and then be recognized by the cytosolic cargo receptors such as NDP52, OPTN, or p62, and bind to ATG8/LC3, delivering the bacteria into autophagosomes for autophagic clearance. Otherwise, NDP52 binds to Galectin 8, a cytosolic lectin that detects host glycans on vesicles damaged by *Salmonella* during the process of entering the host cell. The NDP52-Galectin-8 interaction delivers bacteria for autophagic degradation. Galectin-8 can detect a wide variety of vesicle-damaging pathogens in addition to *Salmonella*, e.g., *Shigella* and *Listeria*. **(B)** In *Shigella* and *Listeria* infection, the bacteria can escape from vacuoles to the host cytosol via their bacterial products (e.g., IpaB, IpaC, LLO, or PLCs). The cytosolic *Shigella* and *Listeria* have actin-based motility, contributing to their escape from autophagy. Essential bacterial products (e.g., IcsA, IcsB, ActA, or InlK) are involved in actin-based motility and inhibition/evasion of autophagy. **(C)** In *Legionella* infection, *L. pneumophila* also escapes from vacuoles to the host cytosol via LepB through Type IV secretion system. Cytosolic *L. pneumophila* is recognized by its autophagic machinery; however, *Legionella* delays fusion of the autophagosome with lysosomes until it develops into an acid-resistant form. The acid-resistant *Legionella* can replicate in the acidic autophagolysosome. *Legionella* also interferes with autophagy by using its bacterial effector protein RavZ.

*S. typhimurium* that enter the cytosol are initially coated with polyubiquitinated proteins, and are then detected by the cargo adaptor, NDP52 (Thurston et al., 2009) (Figure 3A). In addition, *S. typhimurium* activates TLR4 signaling pathways, leading to phosphorylation of TBK-1. Through molecular interaction with adaptor proteins Nap1 and Sintbad, TBK-1, an important signaling molecule for regulation of TIR domain-containing adapter-inducing IFN-β (TRIF)-dependent IRF3 signaling (Yuk and Jo, 2011), is recruited to NDP52, and it phosphorylates OPTN on Ser177, another autophagic receptor (Thurston et al., 2009; Wild et al., 2011). Phosphorylated OPTN has an enhanced ability to interact with the autophagic LC3 protein, driving bacteria toward the autophagic machinery and elimination by xenophagy activation (Thurston et al., 2009; Wild et al., 2011). A more recent study revealed that NDP52 selectively and preferentially interacts with LC3 isoform C (LC3C) through its non-canonical LC3C-interacting region (CLIR) domain structure. Notably, this interaction between LC3C and NDP52 is involved in the recruitment of all ATG8 family members to cytosolic bacteria and successful elimination of *S. typhimurium* (von Muhlinen et al., 2012).

Another cargo adaptor, p62/SQSTM1, is recruited by polyubiquitin-decorated *S. typhimurium* for the xenophagic control of bacteria (Zheng et al., 2009). NBR1 is a cargo adaptor that has a similar domain structure containing an N-terminal PB1 domain, a LIR motif (interacting with LC3 proteins), and a C-terminal UBA domain which interacts with ubiquitin (Kirkin et al., 2009; Lamark et al., 2009). It is known to interact with p62 to form oligomers, it is recruited to polyubiquitinated cargos and degraded by autophagy processes (Kirkin et al., 2009; Lamark et al., 2009). However, it is not known whether NBR1 is involved in *Salmonella* infection. Determining whether it plays a role in the autophagic clearance of intracellular bacteria and whether it can co-operate with other cargo receptors including p62 and NDP52 would be of interest.

In *Salmonella* infection, bacteria initiate an early state of intracellular amino acid deprivation, which is induced by host membrane damage, suggesting that xenophagy is activated by a metabolic switch induced by amino acid starvation (Tattoli et al., 2012). In addition, diacylglycerol (DAG)-induced and ubiquitin-independent autophagy has been reported in host defense against *Salmonella*. DAG, a lipid second messenger generated by phospholipase D, is associated with autophagy-targeted *Salmonella* and is required for antibacterial autophagy through protein kinase Cδ signaling (Shahnazari et al., 2010). Recent studies have also revealed a novel role of cytosolic lectin Galectin 8 (LGALS8) in detecting bacterial invasion through binding to host glycans during invasion by *Salmonella* and *Shigella*. LGALS8 recruits NDP52 (CALCOCO2) to activate antibacterial autophagy (Thurston et al., 2012).

## Antibacterial Autophagy in *Shigella* Infection

*Shigella* is an invasive bacterium that exploits a harmful niche enabling it to replicate inside host cells. During a *Shigella* infection, the bacterium uses an array of pathogenic strategies including; induction of macrophage cell death, a massive inflammatory response, which results in subsequent infection, multiplication within epithelial cells, disruption of the vacuolar membrane surrounding the bacteria, and movement through promotion of actin polymerization (Ashida et al., 2011).

*Shigella* can manipulate the autophagy pathway through escape from and induction of the host autophagic system. *Shigella* can escape autophagy by secreting IcsB through a TTSS (Figure 3B, left), whereas VirG (a protein for intracellular actin-based motility) induces autophagy via interaction with the autophagy protein ATG5 (Ogawa et al., 2005). Additionally, Shiga toxins induce autophagy in THP-1 cells and human macrophages, and enhance cell death of renal epithelial cells through an autophagy-dependent mechanism. Especially in toxin-sensitive cells especially, those toxins are translocated to the endoplasmic reticulum (ER) and activate calpains and caspase-8 and -3, resulting in the cleavage of the autophagy-related genes ATG5 and Beclin-1 (Lee et al., 2011).

Upon invasion of epithelial cells by *Shigella* the vacuolar membrane fragments ruptured by the bacteria are targeted to the autophagy pathway by recruiting ubiquitin, TNF receptor associated factor 6 (TRAF6), p62, and LC3 (Dupont et al., 2009). Interestingly, guanosine triphosphatase (GTP)-binding protein septin assemblies are recruited to intracytosolic *Shigella*, which they entrap in cage-like structures (Mostowy et al., 2010). Moreover, the cargo adaptors p62 and NDP52 direct *Shigella* to an autophagy pathway that is dependent upon septin and actin (Mostowy et al., 2011). During infection, host-derived pro-inflammatory cytokine TNF-α enhances septin caging and p62-mediated autophagic activity, thereby limiting *Shigella* survival and cell-to-cell spread (Mostowy et al., 2010, 2011). A highly conserved Tectonin domain-containing protein, Tecpr1, plays a major role in antibacterial autophagy, targeting *Shigella* through interaction with ATG5 (Ogawa et al., 2011a). Tecpr1-deficient mouse embryonic fibroblasts (MEFs) have a defect in selective autophagy, which is manifested by accumulation of depolarized mitochondria and miss-folded protein aggregates, and an increased replication of *Shigella* (Ogawa et al., 2011a). Importantly since Tecpr1 offers the fusion of autophagosomes and lysosomes by interacting with ATG12-ATG5 and PtdIns3P (Chen et al., 2012), Tecpr1 may play an important role in triggering autophagy in general (Behrends et al., 2010; Ogawa et al., 2011a). *Shigella flexneri* VirA, which harbors TBC-like dual-finger motifs that exhibit GTPase-activating protein (GAP) activity, is known to direct host Rab1 to inhibit IL-8, and counteract autophagy-mediated host defense in infected cells (Dong et al., 2012). Collectively, these studies indicate that the host defense system and the bacterial tactics against the host autophagic machinery, as well as the immune response may determine the outcome of *Shigella* infection.

## Antibacterial Autophagy in *Listeria* Infection

*Listeria monocytogenes (L. monocytogenes)* is a facultative Gram-positive bacteria and an intracellular pathogen that causes listeriosis. Listeriosis commonly affects pregnant women and people with suppressed immune systems, e.g., those with cancer or HIV (Vazquez-Boland et al., 2001). Intestinal epithelial cells are the primary targets of *L. monocytogenes*. After primary infection of the epithelium, the bacterium translocates to phagocytic cells, such as dendritic cells and macrophages, through M cell-dependent or M cell-independent pathways (Barbuddhe and Chakraborty, 2009; Ogawa et al., 2011b). After internalization by the host cell, *L. monocytogenes* escapes from the phagosome to the cytosol by secreting listeriolysin O (LLO), which is a pore-forming hemolysin (Tweten, 2005; Schnupf and Portnoy, 2007; Birmingham et al., 2008). *L. monocytogenes* in the host cytosol expresses the bacterial protein ActA, which engages the host cell actin machinery, to assist bacterial motility and eventually cell-to-cell spread (Moors et al., 1999; Lambrechts et al., 2008). By spreading from cell-to-cell, *L. monocytogenes* disseminates and expands into other cells or tissues.

*L. monocytogenes* has been reported to induce autophagic responses. During the early phase of (∼2 h of post) *Listeria* infection, autophagy plays a crucial role in the host immune defense in mice (Birmingham et al., 2007; Py et al., 2007). *L. monocytogenes* replicates more efficiently in ATG5-deficient MEFs, compared to wild-type (WT) MEFs, suggesting an essential role for autophagy in inhibition of bacterial growth inside the cells (Birmingham et al., 2007; Py et al., 2007). It has also been reported that *L. monocytogenes* induces autophagy activation in *Drosophila* hemocytes (Yano et al., 2008). Moreover, Zhao et al. (2008) revealed that the autophagy protein ATG5 in phagocytic cells, such as macrophages and neutrophils, is essential for *in vivo* immunity to *Listeria* infection (Zhao et al., 2008).

Several possible mechanisms exist by which *L. monocytogenes* triggers the autophagy pathway; one possibility involves the bacterial components, and another is recognition of bacterial invasive process via cytosolic receptors. LLO, a major virulence factor of *L. monocytogenes*, was reported to be a key component of *L. monocytogenes*-induced autophagy (Birmingham et al., 2007; Py et al., 2007). *L. monocytogenes* lacking LLO failed to induce autophagy, cleavage from LC3 I to LC3 II, and co-localization with LC3. Similarly, LLO-mediated membrane remnants of phagosomal rupture were found to be sufficient to activate autophagy (Meyer-Morse et al., 2010). First, LLO-containing liposomes were shown to be recruited to autophagosomes even in the absence of infection (Meyer-Morse et al., 2010). Second, cytosolic receptors, such as peptidoglycan recognition protein (PGRP)-LE or nucleotide-binding oligomerization domain-containing (NOD) 1, play a role in the positive regulation of autophagy during *Listeria* infection (Yano et al., 2008; Travassos et al., 2010). In *Drosophila*, sensing of peptidoglycan by PGRP-LE is required for the induction of autophagy, which can inhibit intracellular growth of *L. monocytogenes* and induce host survival after *Listeria* infection (Yano et al., 2008). In murine and human cells, both NOD1 and ATG16L are recruited to the membranes of vesicles containing *L. monocytogenes*. Notably, the levels of autophagosome-containing *L. monocytogenes* in NOD1 deficient MEFs were significantly lower, compared with those in NOD1 WT MEFs (Travassos et al., 2010).

As autophagy is essential for inhibiting the intracellular growth of *L. monocytogenes* (Birmingham et al., 2007; Py et al., 2007; Zhao et al., 2008), *L. monocytogenes* has evolved diverse evasion strategies against the host autophagy machinery (Birmingham et al., 2007; Py et al., 2007; Yoshikawa et al., 2009; Dortet et al., 2011; Ogawa et al., 2011b) (Figure 3B, right). *L. monocytogenes* has several bacterial components that negatively regulate host autophagy activation. Phospholipases C (PLCs) from *L. monocytogenes*, such as PI-PLC (encoded by PlcA) and PC-PLC (encoded by PlcB), act synergistically with LLO to lyse phagosomal vesicles to promote invasion into the host cytosol. PLCs, however, inhibit host autophagy induced by *L. monocytogenes* (Birmingham et al., 2007; Py et al., 2007). Additionally, ActA, a *L. monocytogenes* surface protein, is involved in intra- and inter-cellular motility enabling escape from autophagy (Dortet et al., 2011; Ogawa et al., 2011b). The ability of the ActA protein to induce recruitment of the Arp2/3 complex and Ena/VASP, contributes to the bacterial ability to evade host autophagic recognition (Yoshikawa et al., 2009). Thus, *L. monocytogenes* lacking ActA is not able be recruited to the Arp2/3 complex and Ena/VASP, it instead becomes ubiquitinated, bind to p62 and LC3, and finally undergoes autophagic clearance (Yoshikawa et al., 2009). Another *L. monocytogenes* surface protein, InlK, acts similarly to ActA (Dortet et al., 2011). Moreover, *L. monocytogenes* lacking ActA showed increased expression of InlK, enabling comparable intracellular survival, similar to WT bacteria. Thus, InlK has a redundant function in *L. monocytogenes* lacking ActA, by replacing ActA and enabling the bacteria to escape autophagic clearance (Dortet et al., 2011). Collectively, these studies indicate that *L. monocytogenes* has dual autophagy regulation mechanisms. While autophagy activation via LLO is as an important defense mechanism against infection, *Listeria* has evolved several evasion mechanisms involving various virulence factors, such as PLCs, ActA, and InlK.

## Antibacterial Autophagy in *Legionella* Infection

*Legionella pneumophila (L. pneumophila)*, although usually found in freshwater protozoa and amebae, is an accidental infectious pathogen that can replicate in alveolar macrophages in the human lung, and especially in immune compromised patients (Dubuisson and Swanson, 2006; Joshi and Swanson, 2011). *L. pneumophila* resides within vacuoles that have features typical of autophagolysosomes, containing the autophagy-related protein ATG8/LC3, lysosomal-associated membrane protein 1 (LAMP1), and the lysosomal acid hydrolase cathepsin D (Dubuisson and Swanson, 2006; Joshi and Swanson, 2011). Notably, the biogenesis of *L. pneumophila*-harboring vacuoles is similar to the formation of autophagosomes. For example, the ER is one source of these two vacuoles, as are the *L. pneumophila* vacuole and the autophagosomal membrane (Joshi and Swanson, 2011). Moreover, this pathogen continuously replicates within acidic lysosomal vacuoles in macrophages, and inhibits immediate delivery to the lysosomes, thus persisting in immature autophagosomal vacuoles (Amer and Swanson, 2005; Joshi and Swanson, 2011). Subsequent secretion of Type IV effectors, including LepB, causes delayed maturation of autophagosomes, and may provide sufficient time for inducing acid resistance and other traits within the autophagolysosomes (Joshi and Swanson, 2011) (Figure 3C).

Several host defense mechanisms, including apoptosis, autophagy, and inflammasome-associated cell death, are thought to form part of the host defense against *L. pneumophila* infection (Swanson and Molofsky, 2005; Banga et al., 2007). *L. pneumophila*-mediated inflammasome activation and pyroptotic cell death is likely to be linked to the autophagy pathway through a mechanism involving the cytoplasmic translocation of flagellin, and its detection via Naip5, a NOD-like receptor (NLR) adaptor protein of the inflammasome (Dubuisson and Swanson, 2006). *In vitro* studies, including treatment of A/J mouse peritoneal macrophages with 2-deoxy-d-glucose, support the role of autophagy in inhibiting the intracellular replication of *L. pneumophila* (Matsuda et al., 2009). *In vivo* studies using the ATG9 mutant *Dictyostelium discoideum* show a critical defect in phagocytosis and clearance of *L. pneumophila*, as well as in growth and development, indicating an important role for autophagy in protection during *L. pneumophila* infection (Tung et al., 2010). Recent studies have revealed a mechanism by which the *L. pneumophila* effector protein RavZ inhibits autophagy by functioning as a deconjugating enzyme that targets ATG8/LC3 proteins attached to phosphatidylethanolamine on autophagosome membranes (Choy et al., 2012) (Figure 3C). Although *Legionella* RavZ can inhibit autophagy by irreversibly inactivating ATG8/LC3 proteins during infection (Choy et al., 2012), whether RavZ-mediated inhibition of autophagy could affect any phenotype of host cells remains to be determined.

## Concluding Remarks

To conclude, the data to date indicate that xenophagy functions selectively target intracellular bacteria through autophagic receptors including SQSTM1/p62, NDP52, OPTN, and NBR1. Cytosolic access of intracellular bacteria or their components, from bacterial vacuoles, initiates the formation and ubiquitination of protein aggregates. During mycobacterial infection, cytosolic sensing of extracellular Mtb-DNA activates ubiquitin-mediated selective autophagy that targets Mtb in an ESX-1 system-dependent manner. However, the cytosolic sensing of Mtb and IRF3-dependent type I IFN signaling are likely to be associated with the pathogenesis of tuberculosis, because IRF deficiency leads to a more protective phenotype against long-term Mtb infection in mice. Whatever the autophagic stimuli, the induction of autophagy by IFN-γ, vitamin D, and TLR ligands is of paramount importance for the elimination of intracellular Mtb in macrophages. In *Salmonella* infection, cytosolic bacteria from damaged SCVs are coated with ubiquitin and recruited to the cargo receptor p62, which interacts with the autophagic machinery. In *Shigella* infections, several cargo receptors, including p62, NDP52, and Tecpr1, contribute to antibacterial autophagy targeting of *Shigella*. *L. monocytogenes* exhibits a dual regulatory function in autophagic regulation through its bacterial components or by modulating host-originated proteins as follows: (1) positive regulation via the virulence factor LLO and host cytosolic receptors, NOD1 or PGRP-LE; (2) negative regulation through *Listeria*-derived components, such as PlcA, PlcB, ActA, and InlK. Curiously, *Legionella* can persist and replicate in immature autophagosomal vacuoles. The *Legionella* effector RavZ was found to subvert host autophagy through delipidation and inactivation of ATG8/LC3. Regardless of the intracellular bacterial strain, host autophagic clearance systems and bacterial manipulation of the host autophagic machinery may determine the outcome of intracellular bacterial infection. Further studies are needed to elucidate the role of bacterial effectors in manipulating host autophagy and to clarify the pathogenesis of intracellular bacterial infections. We believe that this will facilitate the development of innovative treatments for such bacterial infections.

## Conflict of Interest Statement

The authors declare that the research was conducted in the absence of any commercial or financial relationships that could be construed as a potential conflict of interest.
